# Inferring the Potential Distribution of an Emerging Rickettsiosis in America: The Case of *Rickettsia parkeri*

**DOI:** 10.3390/pathogens10050592

**Published:** 2021-05-13

**Authors:** David A. Moo-Llanes, Ana C. Montes de Oca-Aguilar, Dora Romero-Salas, Sokani Sánchez-Montes

**Affiliations:** 1 Centro Regional de Investigación en Salud Pública (CRISP), Instituto Nacional de Salud Pública (INSP), Tapachula, Chiapas 30700, Mexico; davidmooll@gmail.com; 2 Centro de Investigaciones y de Estudios Avanzados, Mérida, Yucatán 97310, Mexico; ana.montesdocaa@gmail.com; 3 Facultad de Medicina Veterinaria y Zootecnia, Universidad Veracruzana, Veracruz 91710, Mexico; 4 Facultad de Ciencias Biológicas y Agropecuarias, Región Tuxpan, Universidad Veracruzana, Tuxpan de Rodríguez Cano, Veracruz 92870, Mexico; 5 Centro de Medicina Tropical, División de Investigación, Facultad de Medicina, Universidad Nacional Autónoma de Mexico, Ciudad de Mexico 06726, Mexico

**Keywords:** vector-borne disease, ticks, ecological niche modeling, *Rickettsia parkeri*, America

## Abstract

Tick-borne rickettsioses represent a severe public health problem that has increased in recent decades by several activities that place human populations in contact with a wide range of vectors. In particular, *Rickettsia parkeri*, an eschar-associated spotted fever agent, represents an emerging pathogen that has been gradually identified throughout America. In the present work, we compiled an occurrence database of these bacteria, as well as its vectors, in order to identify the potential distribution of these bacteria and to detect the risk areas where this emerging pathogen may be circulating. The results show the at-risk areas to be broad regions in Central America, on the coast of Venezuela, Colombia, Ecuador, Peru, and Chile, part of Brazil and Argentina, and the greater part of Bolivia, Paraguay, and Uruguay. Particularly, in Mexico, conditions exist for widespread dissemination. Our results must be considered for the establishment of active acarological surveillance in previously unsampled areas, as well as the establishment of prevention measures for vulnerable populations and risk groups participating in outdoor activities that can place them in contact with this pathogen.

## 1. Introduction

The genus *Rickettsia* harbors around 15 strict intracellular bacterial pathogens that cause several emerging and reemerging diseases of great impact on public health, among which the members of the spotted fevers group (SFG) stand out. The members of the SFG are mainly transmitted by hard ticks of the genera *Amblyomma*, *Dermacentor*, *Ixodes*, and *Rhipicephalus* in America [1]. At least nine *Rickettsia* species of the SFG have recorded in Latin America and the Caribbean, however only five of them have been recorded recognized as human pathogens (*R. africae*, *R. massiliae*, *R. parkeri*, *R. philipi*, *R. rickettsii*). The species with the highest number of confirmed records per country was *R. rickettsii*, the causative agent of the Rocky Mountain spotted fever, followed by *R. parkeri*, the etiological agent of *R. parkeri* rickettsiosis [1,2,3].

*Rickettsia parkeri* was isolated from the Gulf coast tick *Amblyomma maculatum* in the early 1900s in the United States [4]. It was considered a non-pathogenic *Rickettsia* for six decades until 2004, when it was recovered from a human patient with an eschar-associated fever after a tick bite [5]. Subsequently, its epidemiological relevance increased because new human cases began to be detected in the United States, as well as in some countries of the southern cone of America (Uruguay, Argentina, and Brazil), and it is considered an emerging rickettsiosis in America [1,6,7,8,9].

Although the members of the complex *Amblyomma maculatum* (*A. maculatum*, *A. triste*, and *A. tigrinum*) are considered the main vectors of *R. parkeri* in America [2], active acarological surveillance identified 18 species of hard ticks infected with different genetic lineages of this *Rickettsia* species in eight countries. This includes the human pathogen *R. parkeri* strain Atlantic Rainforest in *A. ovale* and *A. aureolatum* [10,11], and others whose pathogenicities are still unknown, such as *R. parkeri* strain NOD39 in *A. nodosum* and *R. parkeri* strain Black Gap in *Dermacentor parumapertus* [1,12,13,14]. *Rickettsia parkeri* is recorded from the United States to Argentina, its distribution shows a discontinuous pattern, with regions absent from records probably due to the lack of surveillance, even though several vector species are included in the local faunistic inventories. These gaps in the knowledge of the real distribution of the species have important epidemiological implications because we do not know the areas where active transmission of this microorganism can occur presently. In addition, the increase in human activities (e.g., hunting, eco-tourism, expansion of sites over wilderness areas) could strongly impact the distribution and determinants of the disease caused by this species. One of the aspects that attracts interest is that the patterns of SF cases in humans have been recorded from the United States to Argentina and Uruguay, and thus far, no human case has been reported in intermediate regions where there are records of ticks infected with *R. parkeri* (e.g., Mexico, Belize, Colombia, and Peru) [14,15,16,17].

New technological tools exist that are based on algorithms that associate occurrences of any species with the environmental conditions that estimate and project potential geographic distributions of a variety of infectious diseases [18]. The Ecological Niche Model (ENM) is frequently used to analyze and predict spatial patterns and the distribution of vector-borne diseases. Multiple abiotic and biotic factors have been associated with the ecological niche as precipitation, temperature, altitude, latitude, physical barriers, and host distribution and abundance [19,20]. ENMs used in emerging diseases or invasive species, such as in the case of the vector *Aedes albopictus* [21], have also been used to validate ecological niche models of the same species in other periods [22]. In that context, and due to the importance of *R. parkeri* as an emerging rickettsiosis agent in America, the goal of this study was to predict the potential geographic distribution of *R. parkeri*, using the occurrence data of ticks infected and ENM theory in America to implement surveillance and vector control measures in the different areas.

## 2. Results

A total of 1479 candidate models were built for this species, while 1295 models were statistically significant. Finally, 10 candidate models met the statistically significant model omission rate and AICc criteria. The best candidate model was a model with set environmental predictors (set 2), regularization multiple (N = 4), feature classes (product), partial ROC (N = 1.06), omission rate 5% (N = 0.03), AICc (N = 2623.39), Delta AICc (N = 0.00), AICc weight (N = 0.81), and number parameters (N = 5) (Table 1).

**Figure 1:**
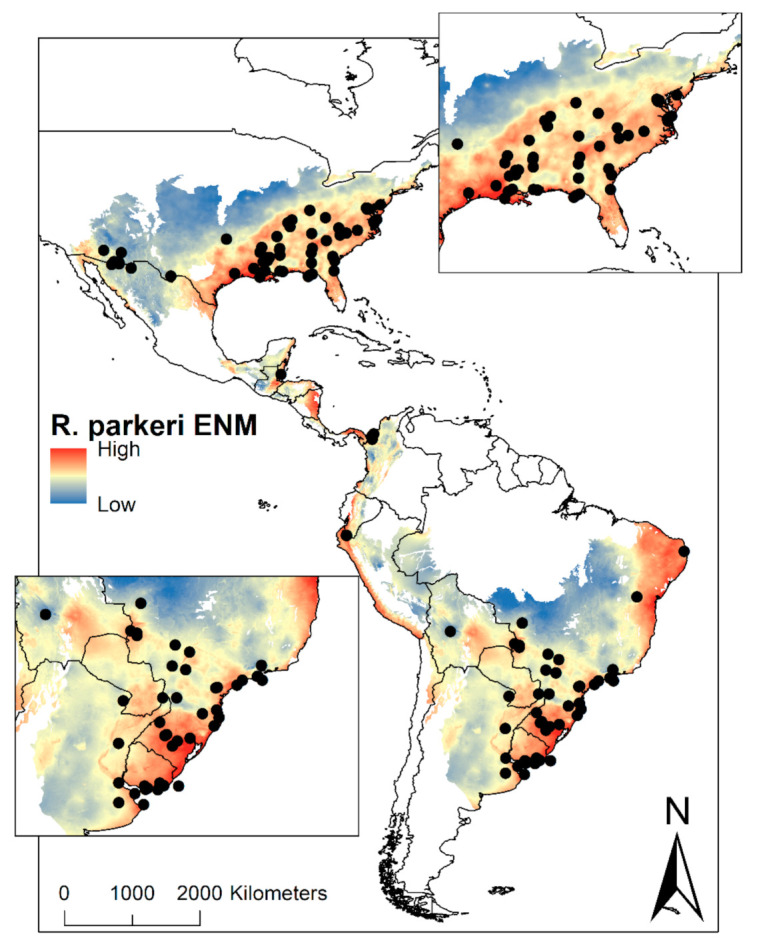
Ecological niche modelling (ENM) of *R. parkeri* in America. On the maps, the suitability of *R. parkeri* from highest to lowest can be visualized. The black dots represent the occurrences of ticks infected with *R. parkeri*. In the upper right box, a zoom in the United States region can be visualized, while in the lower-left box, the zoom of South America can be visualized.

The size of the M region (a set of areas that are accessible to the species) for *R. parkeri* was 22,819,910 km^2^, distributed between United States–Mexico through Central America into South America (Colombia, Venezuela, Ecuador, Peru, Brazil, Bolivia, Paraguay, Uruguay, Argentina, and Chile). Ecological Niche Modelling (ENM) for *R. parkeri* was suitable between 0.08–0.82. The ENM was predicted the potential geographic distribution for the United States and Mexico to Chile and Argentina, except for Central Mexico, Venezuela, Guyana, Suriname, French Guiana, part of Colombia, and Brazil. ENM of *R. parkeri* was also presented in Ontario, Canada. For the United States, the species was predicted in 22 states and 20 states for Mexico. Finally, the ENM was projected in Central America, the coast of Venezuela, Colombia, Ecuador, Peru, and Chile, part of Brazil and Argentina, and the greater part of Bolivia, Paraguay, and Uruguay (Figure 1).

**Figure 2:**
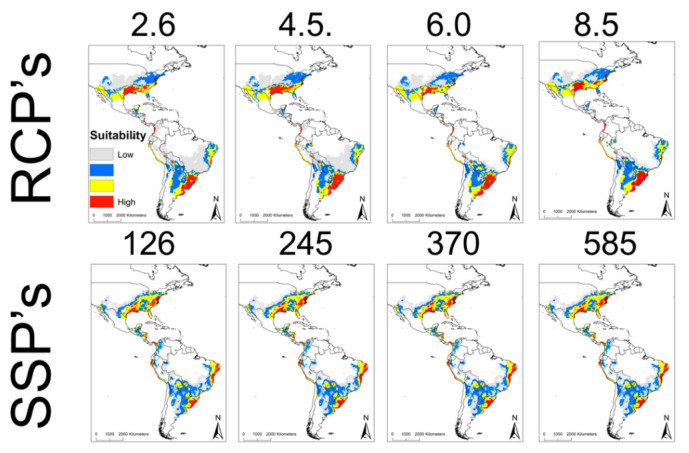
Ecological niche modeling in climate change scenarios of *R. parkeri* in America. The upper panel represents the RCP’s scenarios, while the lower panel represents the SSP’s scenarios. The suitability of the different models is observed from lowest to highest (red). In both scenarios (RCP’s and SSP’s), foci of *R. parkeri* distribution can be observed in the United States and a large part of Brazil, Uruguay, and Argentina.

In the future, all the projections of the climate change scenarios (representative concentration pathways (RCPs) and shared socioeconomic pathways (SSPs)) presented similar geographic distributions. The different projections in the RCP scenarios (2.6, 4.5, 6.0, and 8.5) are similar, mainly affecting regions in Texas, Louisiana, Arkansas, Mississippi, Alabama, Florida, Georgia, South Carolina, North Carolina, and Virginia in the USA, Sonora, Nuevo Leon, Tamaulipas, Oaxaca, Veracruz, Chiapas in Mexico, Guatemala, Costa Rica, Colombia, Peru, Argentina, Uruguay, and Brazil (Figure 2). However, the projections in the SSP scenarios were much more conservative for the year 2041–2060 in the similar regions of the RCPs, mainly in United States, North of Mexico, Brazil, Argentina, and Uruguay. The current ecological niche overlap of *R. parkeri* with the different RCP scenarios presents lower values than with the SSP scenarios (Table 2). The comparisons between the different RCPs were similar (0.954–0.956), compared to the equality of the SSPs values between them (*D* = 1.000) (Table 2).

**Figure 3:**
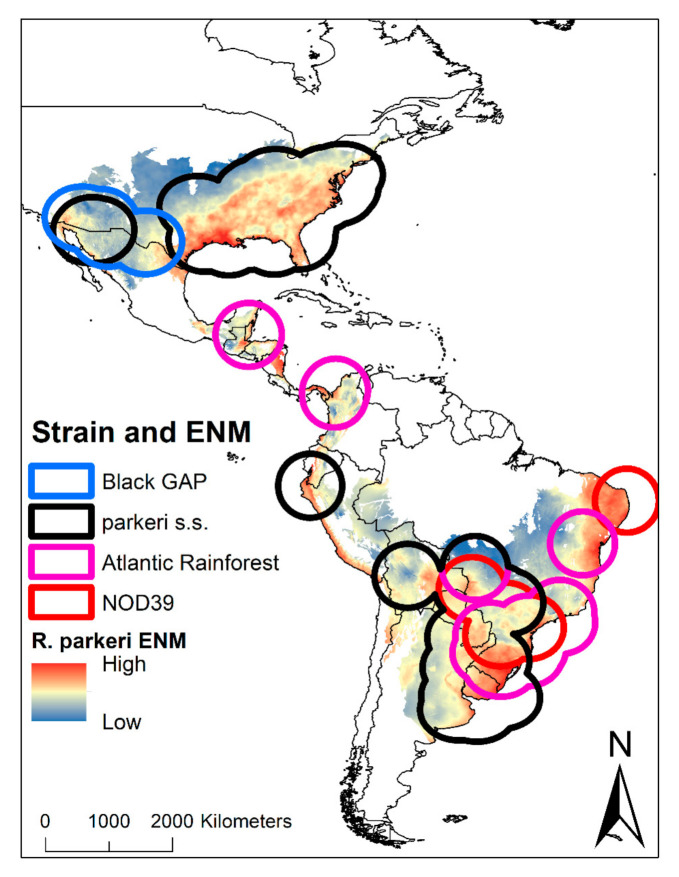
Ecological niche modelling of *R. parkeri* in America with overlapping areas by strains. In the map, the suitability of *R. parkeri* can be observed, as well as the potential buffer areas with the representation of the four strains for America.

The occurrence data of *R. parkeri* were divided into four important strains: Black Gap (N = 4), Parkeri sensu stricto (N = 85), Atlantic Rainforest (N = 22), and NOD39 (N = 6). The Black Gap strain occurs on the United States–Mexico border, compared to the Parkeri sensu stricto strain found in the United States, Mexico, Ecuador, Peru, Brazil, Bolivia, Argentina, Paraguay, and Uruguay. The Atlantic Rainforest strain is located mainly in Mexico, Central America, Colombia, and the intersection of Bolivia, Argentina, Paraguay, and Uruguay. Finally, the NOD39 strain is located in Brazil (Figure 3).

## 3. Discussion

In this study, we show the first database compilation on *R. parkeri* with an ecological analysis of its potential distribution in America. *Rickettsia parkeri* is an emerging pathogen that causes spotted fever rickettsiosis, which has been recorded in several species of ticks of the genus *Amblyomma* (*A. americanum*, *A. aureolatum*, *A. dubidatum*, *A. longirostre*, *A. maculatum/A. triste*, *A. nodosum*, *A. ovale*, *A. parkeri*, and *A. tigrinum*). However, it has also been detected in other hard ticks, such as *Ixodes scapularis*, *Rhipicephalus sanguineus* s.l., *D. parumapertus*, and *D. variabilis* [5,6,7,8,9,10,11,12,23]. Currently, only data from positive infection occurrences have been compared to data from other species of ticks that could be incidental. It is important to consider that several of the tick species incriminated as vectors (e.g., *A. maculatum* and *A. nodosum*), of some of these strains (Parkeri s.s. and NOD39) develop their life cycles in multiple hosts, among which are birds, which in their migratory routes could transfer and disseminate infected ticks from one zoogeographic region to another [2,3,13].

Our current data (Supplementary Table S1; Available in https://doi.org/10.6084/m9.figshare.14452680; accessed on 29 April 2021) present a new perspective of *R. parkeri* in the Americas, showing a wide scope of environmental factors associated with the pathogen’s distribution, as well as the most extensive revision of *R. parkeri* in America (N = 66 published articles). Previous reports have been isolated to nine countries: Argentina (6), Belize (1), Brazil (21), Bolivia (1), Colombia (2), Mexico (2), Peru (1), Uruguay (5) and United States (27) (Figure 1), yet our map shows geographic continuity, making it plausible for the pathogen to have a much wider distribution than expected by sanitary authorities, such as in several unsampled countries of South America (Chile, Paraguay, Ecuador, and Venezuela) and most countries of Central America. Ecological niche modelling of *R. parkeri* was performed for each separate strain: Black Gap, Parkeri sensu stricto, Atlantic Rainforest, and NOD39 (Figure 3). Two of these strains (Atlantic Rainforest and Parkeri s.s.) have a distribution in both the north and south of Latin America; while Black GAP is more restricted to the United States and Mexico border, and NOD39 more restricted to Brazil. It is important to highlight that the Atlantic Rainforest strain has been reported in Argentina and Brazil since 2010, while in the last six years it has been reported in Bolivia and Belize. As of 2018, migrant caravans from Honduras and El Salvador to the Mexico-United States (Available in https://rosanjose.iom.int/SITE/en) border could serve to disperse this strain to new regions in North America. Recently, in Central Mexico, there is no evidence of *R. parkeri*, but we must consider that there is the presence of these vectors in much of the country.

The ecological niche of *R. parkeri* coincides with the ecological niche of several members of the *A. cajennense* complex, a species not yet found with infection [24]. It also coincides with the ecological niche of infected species, such as *A. americanum* [25], and *A. maculatum* [26]. The center of Mexico, where no infection with *R. parkeri* exists, is also highlighted, but with areas suitable for distribution of *I. scapularis* [14,16,27]. Thus far, this is the first article in which different scenarios are evaluated (AR5 and AR6). Previously, ENMs were made for ticks using the RCP scenarios (2050 and 2070). However, none have used the new SSP scenarios [28]. Both scenarios range from conservative projections to very extreme projections. The potential for the increased geographic spread of *R. parkeri* is worrisome, regardless of scenario (RCP or SSP) both in the United States and South America. Therefore, it is important to improve epidemiological control, and surveillance measures must be implemented against *R. parkeri*.

## 4. Materials and Methods

### 4.1. Database and Accessible Area M

We generated a data set according to the ten tick species infected by *R. parkeri*: *A. aureolatum* (2), *A. dubitatum* (3), *A. longirostre* (1), *A. maculatum* (58), *A. nodosum* (4), *A. parkeri* (1), *A. ovale* (20), *A. trigrinum* (2), *A. triste* (22), *D. parumapertus* (4), *Amblyomma* spp (7), and *Dermacentor* spp. (4). The unique occurrences (N = 128) were obtained from 66 manuscripts that reported the presence of *R. parkeri* in Argentina (7), Belize (1), Bolivia (1), Brazil (30), Colombia (4), Mexico (1), Peru (3), Uruguay (12), and the United States (69) (Table S1; Available in https://doi.org/10.6084/m9.figshare.14452680; accessed on 29 April 2021). We eliminated duplicate occurrences and reduced effects of spatial autocorrelation by thinning records with a distance of 5 km between occurrences using the *spThin* R package [29]. We set aside on the data subset and split the remaining occurrences randomly into two subsets: calibration (70%) and testing (30%) using the “random k-fold” method. The latter method partitions the occurrence localities randomly into a user-specified number of (k) bins as described in detail in the protocol of Muscarella et al. [30]. The accessible area M is an important component in the biotic, abiotic and movement diagram [31]. Accessible area M represents the areas to which a species has had access over a relevant time-period because of its movement and colonizing capacities and the structure of barriers and distances [30]. We constructed an accessible area M used 200 km radius buffer around each point to extend the limits of the calibration region. This buffer was subsequently overlain on the ecoregion shapefile of World Wildlife Fund [19,32].

### 4.2. Bioclim Variables

Fifteen (out 19) Bioclim variables (1970–2000) were used to construct ENMs. The 15 layers were downloaded from the WorldClim database version 2.0 [33], excluding those that combined temperature (Bio8 and Bio9) and precipitation (Bio18 and Bio19) owing to known artifacts, consistent with Escobar et al. [34]. We used three sets of environmental predictors (Table 3) [22]: set 1: 15 variables from WorldClim; set 2: variables used for the construction of ENMs of species general included species of medical importance [19,35]; set 3: a jackknife process in MaxEnt used to select distinct sets of variables that contributed mostly to models (>90%) [19,22]. The ENMs were projected for the current period (1970–2000) and two scenarios: RCPs (representative concentration pathways) for year 2050 and SSPs (Shared socioeconomic pathways) for year 2041–2060. ENMs were projected using the four RCPs from the Fifth Assessment Report (AR5), representing the lowest to highest estimated greenhouse gas emissions: 2.6 (>430 ppm CO_2_), 4.5 (580–720 ppm CO_2_), 6.0 (720–1000 ppm CO_2_), and 8.5 (>1000 ppm CO_2_). Finally, ENMs were modelled using the four SSPs from the Six Assessment Report (AR6). These SSPs evaluated the four different ways, i.e., SSP126 (445.6 ppm CO_2_), SSP245 (602.8 ppm CO_2_), SSP370 (867.2 ppm CO_2_), and SSP585 (1135.2 ppm CO_2_), the world might evolve in the absence of climate policy and how different levels of climate change mitigation could be achieved when the mitigation targets of RCPs are combined with SSPs. For the climate model, we obtained the variables at the spatial resolution of 2.5 min (approximately 5 × 5 km per pixel), which is an adequate coarse resolution at which climate influences species distributions [36].

### 4.3. ENM

The ENM was constructed using the *kuenm* package in R software based in MaxEnt [24,37]. We created 1479 candidate models by combining three sets of bioclimatic variables, 17 values of regularization multiplier (0.1–1 with intervals of 0.1, 2–6 with intervals of 1, and 8 and 10), and all 29 possible combinations of five feature classes (linear = l, quadratic = q, product = p, threshold = t, and hinge = h) [24]. Regularization multiplier forces have many coefficients that are zero; therefore, a greater deviation from zero corresponds to a greater penalty in the model fit. The feature classes are the response categories that the data can belong to using n binary characteristics [37,38]. The candidate model performance and best model were selected according to the criteria proposed by Cobos et al. [24], as follows: AICc, Delta AICc, and Weight AICc. The final model was based on the criteria established by Cobos et al. [24]. After model calibration, we created final models with the selected parameter values, using all occurrences after the corresponding thinning process, with 10 bootstrap replicates with logistic outputs. To further visualize the effects of uncontrolled model projections on the future climate, we also developed predictions allowing predictions in novel climates (i.e., extrapolation with activated clamping) for a transferred-use type of extrapolation with clamping to the RCPs and SSPs [38]. We evaluated the ecological niche overlap using the Schoener *D* index in the ENMTools software with a range from 0 (no overlap) to 1 (total overlap) [39]. Finally, a 200 km radius buffer was created around each point to extend the limits of the occurrence points of four strains of *R. parkeri.*

## 5. Conclusions

The findings of the present study have important implications in public health because they reveal the presence of a zoonotic agent that circulates in several tick species in different localities across the Americas. Given that the members of this group have cross-reactivity in the serological tests, it is possible the patients may be infected by this species that presents with a milder rickettsiosis than those infected by *R. rickettsii*, the etiological agent of Rocky Mountain Spotted Fever [1,2]. For this reason, it is essential to perform an intentional search for patients infected with this eschar-associated pathogen, as well as reinforce acarological surveillance in regions with the potential presence of the pathogen to establish the degree of exposure for the human population.

## Figures and Tables

**Table 1:** Model performance under optimal parameters using regularization multiplier (RM).

Best Models	RM	FC *	p.ROC	O.rate 5%	AICc	∆AICc	AICc.W	#
1th	4	p	1.06	0.03	2623.39	0.00	0.81	5
2th	4	qp	1.06	0.03	2623.42	0.03	0.43	5
3th	5	lqpt	1.06	0.00	2624.43	1.03	0.14	7
4th	5	lqpth	1.06	0.00	2624.43	1.03	0.10	7
5th	5	lpt	1.05	0.00	2624.51	1.12	0.16	7
6th	5	lpth	1.05	0.00	2624.51	1.12	0.11	7
7th	6	lqpt	1.05	0.00	2625.01	1.62	0.06	5
8th	6	lqpth	1.05	0.00	2625.01	1.62	0.05	5
9th	6	lpt	1.05	0.00	2625.02	1.63	0.06	5
10th	6	lpth	1.05	0.00	2625.02	1.63	0.05	5

Features classes (FC), partial ROC (p.ROC), omission rate 5% (O.rate 5%), Akaike Information Criterion corrected (AICc), Delta Akaike Information Criterion corrected (∆AICc), Akaike Information Criterion corrected weight (AICc.W) and number parameters (#) for modeling ecological niche for *Rickettsia parkeri*. * l = Linear; q = Quadratic; t = Threshold; h = Hinge; p = Product.

**Table 2:** Niche overlap values using Schoener’s *D* index between all different times for *R. parkeri* in America. Niche overlap is represented from 0 (no overlap) to 1 (full overlap).

Times	RCP2.6	RCP4.5	RCP6.0	RCP8.5	SSP126	SSP245	SSP370	SSP585
**Current**	0.836	0.841	0.839	0.836	0.964	0.964	0.964	0.964
**RCP2.6**		0.956	0.954	0.955	0.834	0.834	0.834	0.834
**RCP4.5**			0.95	0.96	0.842	0.842	0.842	0.842
**RCP6.0**				0.954	0.839	0.839	0.839	0.839
**RCP8.5**					0.837	0.837	0.837	0.837
**SSPS126**						1.000	1.000	1.000
**SPSS245**							1.000	1.000
**SPSS370**								1.000

**Table 3:** Set of Bioclim variables used to the construction of the ecological niche modeling for *Rickettsia parkeri.*

Bioclim Variables	Code	set1	set2	set3
Annual Mean Temperature	Bio01	**X**	**X**	**X**
Mean Diurnal Range	Bio02	**X**		
Isothermality (BIO2/BIO7) (×100)	Bio03	**X**		
Temperature Seasonality (standard deviation × 100)	Bio04	**X**	**X**	**X**
Max Temperature of Warmest Month	Bio05	**X**	**X**	
Min Temperature of Coldest Month	Bio06	**X**	**X**	**X**
Temperature Annual Range (BIO5-BIO6)	Bio07	**X**	**X**	**X**
Mean Temperature of Warmest Quarter	Bio10	**X**		
Mean Temperature of Coldest Quarter	Bio11	**X**		
Annual Precipitation	Bio12	**X**	**X**	
Precipitation of Wettest Month	Bio13	**X**	**X**	
Precipitation of Driest Month	Bio14	**X**	**X**	**X**
Precipitation Seasonality (Coefficient of Variation)	Bio15	**X**	**X**	**X**
Precipitation of Wettest Quarter	Bio16	**X**		
Precipitation of Driest Quarter	Bio17	**X**		

## Data Availability

All data are publicly available in the manuscript text. Occurrence records of *R. parkeri* in America were deposited via the Figshare repository in https://doi.org/10.6084/m9.figshare.14452680.

## Supplementary Materials

The following are available online at: https://doi.org/10.6084/m9.figshare.14452680, Table S1: Occurrence records of *R. parkeri* in America.
